# Progenitor resilience and the early onset of chronic lung diseases: NHLBI workshop report

**DOI:** 10.1093/ajrcmb/aanag107

**Published:** 2026-05-27

**Authors:** SeungHye Han, Anny Xiaobo Zhou, Amanda M Jamieson, Andrew A Wilson, Bin Zhou, Daniel J Weiss, Dianhua Jiang, Frank McKeon, Harold Chapman, Helen Miranda, Herbert B Schiller, Jayaraj Rajagopal, Jeong H Yun, Jianwen Que, John F Engelhardt, Maria C Basil, Micha Sam Brickman Raredon, Mingxia Gu, Moumita Ghosh, Preetish Kadur Lakshminarasimha Murthy, Purushothama Rao Tata, Ruobing Wang, Shawn Davidson, Susan M Majka, Yohannes Tesfaigzi, Christian R Gomez, Qing Lu, Jose Ordovas-Montanes, Ravi Kalhan

**Affiliations:** Division of Pulmonary and Critical Care Medicine, Northwestern University, Chicago, IL, United States; Department of Medicine, University of Virginia School of Medicine, Charlottesville, VA, United States; Department of Molecular Microbiology and Immunology, Brown University, Providence, RI, United States; Pulmonary, Allergy, Sleep and Critical Care Medicine, Boston University, Boston, MA, United States; Center for Excellence in Molecular Cell Science, Chinese Academy of Sciences, Shanghai, China; Department of Medicine, University of Vermont, Burlington, VT, United States; Department of Medicine, Cedars Sinai Medical Center, Los Angeles, CA, United States; Department of Biology and Biochemistry, University of Houston, Houston, TX, United States; Pulmonary and Critical Care Division, University of California, San Francisco, CA, United States; Department of Genetics and Genome Sciences, School of Medicine, Case Western Reserve University, Cleveland, OH, United States; Research Unit Precision Regenerative Medicine, Helmholtz Munich, Comprehensive Pneumology Center Munich (CPC-M), Member of the German Center for Lung Research (DZL), Munich, Germany; Department of Internal Medicine, Harvard Medical School, Massachusetts General Hospital, Boston, MA, United States; Pulmonary and Critical Care Medicine, Department of Medicine, Brigham and Women’s Hospital, Harvard Medical School, Boston, MA, United States; Department of Medicine, Columbia University, New York, NY, United States; Division of Pulmonary, Allergy and Critical Care Medicine, University of Alabama at Birmingham, Birmingham, AL, United States; Department of Medicine, University of Pennsylvania, Philadelphia, PA, United States; Department of Anesthesiology, Yale School of Medicine, New Haven, CT, United States; Department of Anesthesiology and Perioperative Medicine, David Geffen School of Medicine, Broad Stem Cell Research Center, University of California, Los Angeles, Los Angeles, CA, United States; Division of Pulmonary, Allergy and Critical Care Medicine, Department of Medicine, University of Colorado, Anschutz Medical Campus, Aurora, CO, United States; Department of Pharmacology and Regenerative Medicine, University of Illinois Chicago, Chicago, IL, United States; Department of Cell Biology, Duke University, Durham, NC, United States; Division of Pulmonary Medicine, Boston Children’s Hospital, Boston, MA, United States; Division of Pulmonary and Critical Care Medicine, Northwestern University, Chicago, IL, United States; Division of Pulmonary and Critical Care, National Jewish Health, Denver, CO, United States; Pulmonary and Critical Care Medicine, Department of Medicine, Brigham and Women’s Hospital, Harvard Medical School, Boston, MA, United States; Division of Lung Diseases, National Heart, Lung, and Blood Institute, National Institutes of Health, Bethesda, MD, United States; Division of Lung Diseases, National Heart, Lung, and Blood Institute, National Institutes of Health, Bethesda, MD, United States; Division of Gastroenterology, Hepatology, and Nutrition, Boston Children’s Hospital, Boston, MA, United States; Program in Immunology, Harvard Medical School, Boston, MA, United States; Broad Institute of MIT and Harvard, Cambridge, MA, United States; Harvard Stem Cell Institute, Cambridge, MA, United States; Ragon Institute of MGH, MIT and Harvard, Cambridge, MA, United States; Division of Pulmonary and Critical Care Medicine, Northwestern University, Chicago, IL, United States

**Keywords:** lung stem cell, resilience, chronic lung disease

## Abstract

Lung function peaks in young adulthood and declines with age. Studies suggest that this trajectory may be modifiable, potentially enhancing lung health and resilience to prevent or delay the onset of chronic lung disease. Although significant progress has been made in identifying the lung stem/progenitor cell populations involved in lung repair and homeostasis, their regulation and potential for manipulation to promote respiratory health and resilience remain elusive. To explore these issues, the NHLBI organized a virtual workshop on November 7-8, 2024, that aimed to cover the current scientific landscape; address critical research gaps; and identify challenges, opportunities, and key research questions related to chronic lung disease, with a focus on lung stem/progenitor resilience and its applications in the early diagnosis, prevention, and treatment of chronic lung diseases. This report summarizes the scientific presentations, discussions, and recommendations from the workshop.

Chronic lung diseases, such as chronic obstructive pulmonary disease (COPD) and idiopathic pulmonary fibrosis (IPF), impose significant global health challenges. The prevalence of these diseases is alarmingly high worldwide, with millions of individuals affected.^1^^,^^2^ Factors such as aging, genetic predisposition, and environmental exposures including smoking, air pollution, and occupational hazards represent high risk factors for chronic lung disease. Despite recent advances in our understanding of the pathogenesis of chronic lung disease, there is currently no treatment available to reverse the course of the disease. Efforts to focus on prevention and early diagnosis to slow progression are therefore emerging as effective and proactive strategies.

Lung stem/progenitor cells promote lung repair and regeneration by differentiating into various lung cell types, thereby repairing damaged tissue and promoting tissue resilience. Advances in experimental methods have led to the identification of multiple epithelial cell populations with stem cell capabilities in the lung, some of which expand in response to lung injury and in diseased lungs.^3^ However, the precise role of these cells in maintaining respiratory health and their potential as early diagnostic and/or therapeutic approach to enhance respiratory health and resilience remain underexplored.

The NHLBI of the NIH convened a virtual workshop, titled Progenitor Resilience and the Early Onset of Chronic Lung Diseases, on November 7-8, 2024. The goal of the workshop was to bring together pulmonary medicine researchers, stem/progenitor cell biologists, and technology developers to understand the current state of the science and to identify critical research gaps, challenges, opportunities, and key research questions related to resilience in chronic lung disease. Special emphasis was placed on lung progenitor cell resilience and its role in the early diagnosis, prevention, and treatment of chronic lung diseases. In addition, this workshop sought to foster collaboration across basic science, clinical research, and industry in the focus area. This report highlights the scientific presentations, discussions, and future research opportunities identified during the workshop.

## Respiratory health and resilience

Human lung development continues after birth, reaching a peak in functionality in young adulthood. The peak lung function varies among individuals based on factors such as sex, height, race, genetics, and prenatal and early life exposures. With aging, lung function gradually declines, and some individuals may develop chronic lung disease (Figure 1). Some people experience an accelerated decline in lung function, making them more susceptible to chronic lung disease.^4^ Conversely, some experience a slow decline in lung function, even after respiratory infections, demonstrating resilience against disease development. The rate of decline is known to be associated with factors such as smoking, air pollution, obesity, genetic predisposition, and environmental exposures. Studies suggest that the rate of decline may be modifiable to prevent and/or delay the onset of chronic lung disease.^5-8^

**Figure 1:**
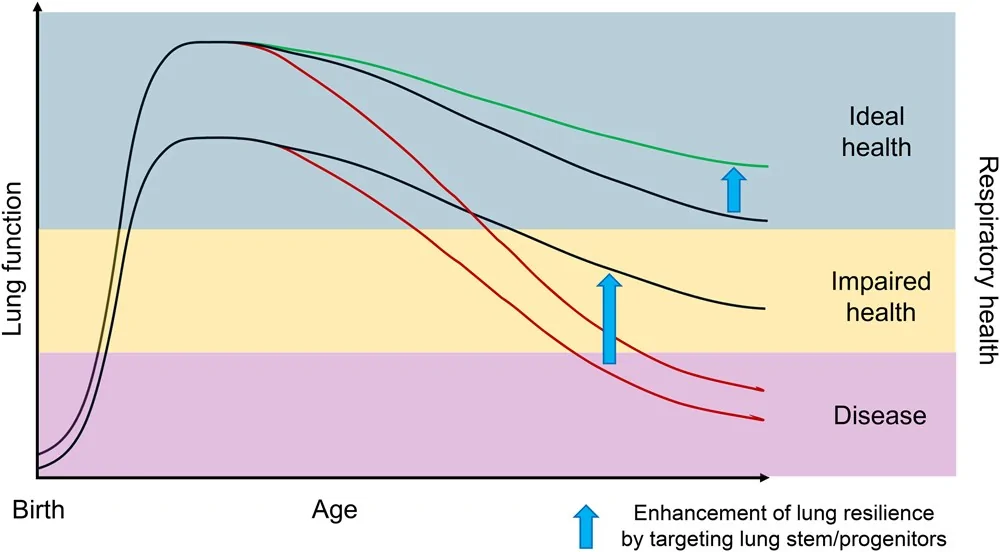
A conceptual model of respiratory health and lung resilience. The human lung continues to develop after birth and reaches its maximum capacity in early adulthood, although this peak varies among individuals. As people age, lung function gradually declines, and both genetic predisposition and environmental exposures influence the rate of this decline. A more rapid decline in lung function may increase susceptibility to disease, while a slower decline or the ability to return to baseline lung function after injury indicates lung resilience. Future research should prioritize understanding the mechanisms that control these lung function trajectories, with the goal of mitigating the “normal” age-related decline (primary prevention) and slowing disease progression (secondary prevention) by promoting respiratory health (blue arrows).

The NHLBI has emphasized the importance of primary prevention of chronic lung disease by promoting respiratory health.^9^ Respiratory health should be viewed as a continuum rather than a simple dichotomy of being disease-free. One proposed approach categorizes the status of respiratory health into ideal health, impaired health, and disease.^10^^,^^11^ Studies show that individuals with impaired respiratory health (lower limit of normal or with respiratory symptoms) are more likely to develop chronic lung disease than those with ideal respiratory health, even after adjusting for risk factors such as smoking and age.^12-15^ These findings support the concept of respiratory health as a continuum and validate approaches to target those with impaired respiratory health and mitigate their decline in lung function by promoting respiratory health.

Unlike the cardiovascular research field, the respiratory community lacks a cholesterol-like biomarker that is central to the causal pathway of disease pathogenesis and thus capable of predicting prognosis, monitoring disease progression, and measuring treatment efficacy. Pulmonary stem/progenitor cells, by definition, have regenerative potential (eg, capacity for self-renewal and differentiation) to repair injured airways and alveolar-capillary respiratory units and thus play an essential role in respiratory health and resilience. Research efforts to date have focused on the identification of pulmonary stem/progenitor cells and their presence/expansion in the context of end-stage disease lungs. However, to understand the mechanistic factors contributing to lung health and resilience, it is critical to understand how these stem/progenitor cells are regulated to maintain lung homeostasis and achieve successful repair after injury. This knowledge could pave the way for the discovery of progenitor-based biomarkers that can predict the early onset of chronic lung disease and assess treatment response and ultimately the discovery of new therapies to reverse disease course. During the workshop, discussants focused on the current understanding of lung stem/progenitor cells, new technologies to study progenitor cell behavior, ongoing efforts to identify biomarkers, therapeutic strategies, and lessons learned from other organ systems.

## Pulmonary progenitor/stem cells in respiratory health and chronic lung disease

Recent advances in experimental technology have enabled the identification of several previously undescribed epithelial cell types, some of which exhibit stem cell properties (eg, self-renewal and differentiation). Comprehensive in vivo and in vitro studies have confirmed that alveolar epithelial type II (AT2) cells are partially committed stem cells that can self-renew and differentiate into alveolar epithelial type I (AT1) cells, which play a critical role in gas exchange.^16^^,^^17^ While several pathways have been proposed to regulate AT2 stem cell behavior during lung homeostasis and postinjury repair,^18^^,^^19^ their potential as therapeutic targets for improving patient outcomes remains uncertain.

During AT2-to-AT1 cell differentiation, an intermediate transitional epithelial cell type emerges.^20-23^ Although rare in adult homeostasis, this intermediate cell type expands during repair after injury and in diseased lungs such as pulmonary fibrosis.^24-26^ Multiple pathways including oxidative phosphorylation, unfolded protein response, transforming growth factor (TGF)–β signaling, p53 pathway, cell adhesion/tight junction, and cellular senescence were enriched in the transitional cell state.^20-23^ The biological cues that drive the expansion of these transitional cells, as well as their functional roles in tissue repair and disease progression, remain areas of active investigation. Notably, ablation of AT2 cells did not induce the transitional cell state,^27^ whereas ablation of AT1 cells alone was sufficient to induce the transitional cell state.^28^ Furthermore, accumulation of transitional epithelial cells can trigger alveolar transitional fibroblasts, leading to fibrosis.^28^^,^^29^ Conversely, loss of transitional alveolar epithelial cells or fibroblasts resulted in tissue simplification.^28^ This suggests that common repair mechanisms following injury could underlie conditions like emphysema and fibrosis, representing 2 extremes of the same response to damage.

A recent study revealed the role of the mitochondrial-integrated stress response in controlling AT2 stem cell fate.^30^ Mitochondrial dysfunction, specifically the loss of mitochondrial electron transport chain (ETC) complex I in the lung epithelium during development, leads to the postnatal expansion of transitional epithelial cells, resulting in postnatal death of the animals from respiratory failure. Independent of ATP synthesis, a high NADH/NAD^+ ^ratio due to loss of ETC complex I function leads to a pathologically high and persistent activation of the integrated stress response (ISR). This prevents the intermediate transitional cells from differentiating into AT1 cells. Notably, inhibiting the ISR facilitates the differentiation of transitional cells into AT1 cells, even when ETC complex I function is absent.^30^ Aligning with these findings, studies have shown that the ISR inhibitor ameliorates fibrosis in various animal models of pulmonary fibrosis.^31^^,^^32^ The ISR is a conserved signaling pathway that helps cells and organisms adapt to various environmental stresses.^33^ Each of 4 kinases—HRI (heme-regulated inhibitor), PKR (double-stranded RNA-dependent protein kinase), PERK (PKR-like ER kinase), and GCN2 (general amino acid control nonderepressible 2)—senses various stresses, leading to the phosphorylation of the eukaryotic translation initiation factor eIF2 to activate the ISR. Traditionally, ISR activation has been viewed as a mechanism to maintain or restore physiological homeostasis in a cell, with unresolved stress ultimately triggering apoptosis to eliminate damaged cells. This study reveals a previously unrecognized link between mitochondrial NAD^+ ^regeneration capacity, AT2 stem cell fate, and the ISR, providing a novel concept—that the way stem/progenitor cells respond to stress can alter their stem cell capabilities—and offering new opportunities for prevention and therapy.

The discussion also centered around the distal airway progenitor cells, unique in the human lung. While absent in mice, humans have the terminal and respiratory bronchioles (TRBs) before transitioning into alveolar duct, which have been implicated in chronic lung disease.^34-36^ Recently, 2 independent groups identified previously undescribed cell types in the human distal respiratory airways: respiratory airway secretory cells, TRB-specific alveolar type-0 (AT0) cells, and TRB secretory cells (TRB-SCs).^37^^,^^38^ Respiratory airway secretory cells—as defined by Basil and colleagues^38^—encompass the TRB-SC population described by Murthy and colleagues,^37^ as well as more proximal SCGB3A2^+^/SCGB1A1^+ ^secretory cells, whereas SCGB3A2^+^/SFTPC^+ ^AT0 cells represent a distinct population normally restricted to respiratory bronchiole-adjacent alveolar septae. These cell types, identified in human, nonhuman primate, and ferret lungs, exhibit stem cell capacities and may differentiate into AT2, AT1, or TRB-SCs. These findings are consistent with Habermann et al.,^24^ who observed an expansion of *SCGB3A2*^+ ^cells co‑expressing AT2 or AT1 markers in human pulmonary fibrosis. Alterations in these cell populations have also been observed in airway-diseased lungs, including COPD.^38^^,^^39^ Future research should focus on understanding the role of these progenitor cell populations in respiratory pathophysiology and their potential to promote lung resilience.

Additionally, in human proximal airway, a novel airway cell type known as hillock basal stem cells was recently identified,^40^ as plastic airway stem cells that are resistant to physical freezing, viral infection, and exposure to irritants. The function of hillocks in human airway diseases remain elusive yet. Future studies may further characterize the function of hillocks cells and their contribution to airway resilience in various injury models.

Overall, significant progress has been made in defining progenitor cell states and subsets. Future research should focus on elucidating the molecular mechanisms that control these newly discovered lung stem/progenitor cells and how they influence lung health and resilience or disease susceptibility.

## Emerging technologies and model systems to study progenitor cells and resilience

Recent advances in technology and model systems have significantly deepened our understanding of progenitor cells and their roles in tissue resilience. The discussion highlighted cutting-edge tools that enable precise in vivo cell fate mapping and the development of human-specific models, offering new opportunities for therapeutic discovery.

One of the technologies enabling the marked advancement of stem cell biology is in vivo lineage tracing, which allows us to track the stem cell differentiation and the origin of differentiated cells in living animals. In the past, we could only label 1 cell type with a single marker gene and follow its proliferation and differentiation in vivo. Recently, however, a dual reporter system has been developed to label the expression of 2 genes simultaneously. This system enables us to label multiple cell types at the same time or to precisely label a specific cell type co-expressing 2 different marker genes, such as bronchioalveolar stem cells (BASCs).^41^^,^^42^ This system confirmed the in vivo contribution of club cells and BASCs to alveolar repair after severe lung injury and that AT1 cells do not differentiate into AT2 cells in vivo during homeostasis or repair after bleomycin injury and lung growth postpneumonectomy in mice.^43^

The discussion also emphasized the value of nonrodent mammal models to study lung disease and resilience. Although animal studies in rodents have provided important biological insights into the pathophysiology of lung disease and health, anatomical and cellular differences between human and rodent airway structure^44^ pose challenges in the study of certain human lung diseases. For example, due to species-specific differences in conducting airways, cystic fibrosis transmembrane conductance regulator (CFTR) knockout mice do not develop phenotypes similar to those seen in CF patients,^45^ whereas CFTR knockout induces the human CF phenotypes in pigs^46^^,^^47^ and ferrets,^48^^,^^49^ making them more suitable for disease modeling. As noted above, the mouse lung, unlike the human lung, lacks the respiratory bronchioles, which limits the application of the mouse study findings to human lung diseases, including COPD and IPF, which are known to be associated with abnormalities in the respiratory bronchioles. Ferrets, however, possess respiratory bronchioles and exhibit clinical features of human COPD.^50^ A recent study also reports that unlike in mice, bleomycin injury in ferrets induces nonresolving fibrosis and transcriptional changes in the epithelium similar to those seen in human IPF.^51^^,^^52^ In addition, several transgenic ferret models are being developed to enable in vivo cell fate mapping.^49^ Transgenic ferrets are ideal models to study human lung disease, with enhanced translatability for future therapies.

Recent progress of 3D organoid systems has emerged as valuable preclinical in vitro models, in particular with organoids derived from patients’ lung cells or patient-derived induced pluripotent stem cells (iPSC) to study lung disease biology. For example, the patients’ somatic cells–derived iPSC system carries individual patient-specific genetic information with noninvasive renewable supply of cells and is easily amenable to gene editing. Such iPSC-derived organoids maintain the genetics from donors during complex disease modeling, offering a broadly applicable platform for studying progenitor cell biology. Several protocols have been developed to differentiate human iPSCs into airway epithelial cells^53^^,^^54^ or AT2 cells.^55^^,^^56^ An iPSC-based airway culture system includes common and rare airway cell types, including pulmonary ionocytes, that mimic the ion transport defects of cystic fibrosis, providing a platform for pathogen studies, drug screening, and understanding of CF pathology.^54^ Other examples include human iPSC-derived AT2 (iAT2) cell cultures to model chronic lung disease in vitro, incorporating genetic susceptibility and environmental exposure. Transcriptomically similar to human primary AT2s, iAT2s can recapitulate functional features of the patient of origin.^55^ When cultured at an air-liquid interface with exposure to cigarette smoke, iAT2s can model emphysema in vitro.^57^ CRISPR interference of genes associated with COPD by genome-wide association studies in iAT2s elucidated the biological function of the desmosomal component gene *DSP* (desmoplakin) in AT2 cells.^58^

The limitation of 3D culture systems is that iPSC-derived or primary lung epithelial cells may not achieve full maturation or differentiation in the in vitro 3D culture system as in native in vivo lung tissue and that it lacks complex lung microenvironment including extracellular matrix, mechanical properties, and interaction with other cell types such as immune and vascular components. To overcome this at least partially, a human iPSC-derived vascularized lung organoid system was developed^59^ by co-differentiating mesoderm and endoderm lineages from iPSC into vascularized lung organoids consisting of mesenchyme, endothelium, and lung epithelial progenitors. Vascularized lung organoids showed 3D architecture similar to in vivo mouse lungs at E12.5 and cell-type composition similar to human fetal lungs. Furthermore, vascularized lung organoids using iPSC from alveolar capillary dysplasia (ACD) patients with *FOXF1* mutations showed abnormal capillary differentiation, which is the key phenotype of ACD patients.^59^ This system can provide a better understanding of epithelial-endothelial interactions than the traditional organoid culture system. Recently, a computational method to study cell-cell interaction was developed that can be applied to single-cell RNA sequencing atlas or spatial transcriptomic data.^60^ These innovations align with the recent NIH’s initiative to prioritize human-based research and promote new approach methods.

## Efforts to identify progenitor cell-based biomarkers on causal pathways

Identification of biomarkers is critical for early diagnosis and better prognosis of chronic lung diseases. In particular, biomarkers that lie on causal pathways can provide crucial insight into how chronic lung diseases develop and progress: detecting early signs of disease onset, predicting disease course and treatment response, and monitoring disease progression and treatment response. The development of these biomarkers requires a deep understanding of the underlying biological mechanisms, rigorous validation, and standardization to ensure reliability across diverse populations. We are beginning to understand the molecular and metabolic signaling pathways that control lung stem/progenitor cell behavior during lung homeostasis and repair upon injury, including the ISR, Wnt/β-catenin, p53, fibroblast growth factor (FGF), epidermal Growth Factor (EGF), transforming growth factor (TGF)-β, YAP/TAZ, cell division cycle 42 (Cdc42), bone morphogenetic protein (BMP), and Notch.^18^^,^^19^ As our understanding of how lung stem/progenitor cells are regulated advances, it is important to focus our efforts on identifying lung stem/progenitor-based biomarkers on causal pathways, complemented with omics-based screening approaches, that can predict lung health and resilience against the development of chronic lung disease.

Notably, a study has reported that airway epithelial progenitor cells obtained from patients with COPD through endobronchial biopsies exhibit reduced colony-forming efficiency as compared to those without COPD, and the colony-forming efficiency correlates with lung function.^61^ Ongoing cross-sectional and longitudinal studies are investigating airway progenitor basal cell dysfunction in young smokers prior to COPD onset as a potential biomarker, with emphasis on altered bioenergetics and mitochondrial metabolism.

Omics-based methodologies such as genomics, proteomics, metabolomics, and transcriptomics offer powerful tools for biomarker discovery, allowing unbiased screening approaches. For example, approaches integrating advanced single-cell genomics and proteomics have identified protein markers in bronchoalveolar lavage fluid and plasma that correlate with lung function and disease progression in patients with pulmonary fibrosis.^62^ Nonetheless, a recognized limitation of omics data is its descriptive nature with limited causal inference into functional phenotypes. To address this issue, perturb-seq has been applied in various cellular models, which combines CRISPR-based genetic screening with single-cell RNA sequencing to profile the effects of genetic perturbations on genome-wide gene expression.^63^^,^^64^ In this approach, perturbations such as drugs or genetic modifications are applied to the cell or tissue platform (eg, precision-cut lung slices or 3D organoid culture systems), followed by single-cell genomic analyses. Using this strategy, disease trajectories can be mapped in the human lung, and cell states can be correlated to disease stages or lung health. Longitudinal cohort studies that collect tissue samples with detailed clinical data throughout life or at least during disease progression are vital, especially to include healthy normal state and early stage disease, thereby reducing the bias associated with end-stage lung tissue data in the development of biomarkers to predict lung health and resilience.

## Therapeutic approaches

Cell-based therapies, particularly those using mesenchymal stromal cells (MSCs), have been explored for a range of inflammatory conditions including acute respiratory distress syndrome (ARDS) and COPD. Typically, MSCs are harvested from bone marrow or adipose tissue and then expanded in culture. They are usually administered intravenously, where they first pass through the pulmonary capillary bed. These cells do not engraft and are cleared within a few days. They are thought to exert immunosuppressive effects through interactions with immune cells. Although MSC-based cell therapies have shown promising results in preclinical models of acute lung injury, they have yielded mixed results in clinical trials in patients with ARDS with or without COVID-19.^65^ Notably, a recent post hoc analysis of a phase II trial in COPD patients suggested that MSCs might improve lung function in individuals with baseline C-reactive protein levels greater than 4 mg/L.^66^ Future trials should clarify the optimal cell source, manufacturing methods, dosage, route of administration, and appropriate inflammatory profiles of target patient populations. In addition to MSC therapies, emerging strategies such as extracellular vesicle delivery and mitochondrial transfer are being investigated, while efforts to achieve successful engraftment using iPSC-derived epithelial stem cells continue.

Recent insights into lung stem cell regulation are paving the way for innovative therapeutic strategies in preclinical models of lung disease. In particular, research has shown that zinc metabolism is critical for AT2 cell renewal in a sirtuin 1 (SIRT1)–dependent mechanism.^67^ Expression of the zinc transporter gene, *SLC39A8* (ZIP8), was decreased in IPF lungs compared to healthy lungs. Loss of *Slc39a8* was associated with impaired renewal capacity of AT2s and worse fibrosis after bleomycin injury in mice. Zinc-deficient diet exacerbates lung fibrosis, and zinc supplementation reduced fibrosis in bleomycin-induced lung injury models in mice and improved the renewal capacity of AT2 cells via SIRT1.^67^ Interestingly, the expression of NAD^+ ^synthesis enzymes, a central tricarboxylic acid (TCA) cycle metabolite and a key substrate for maintaining the deacetylation activity of SIRT1, was downregulated in AT2 cells from IPF patients, and supplementation with NAD^+ ^precursors, nicotinamide mononucleotide (NMN), or nicotinamide riboside enhanced AT2 cell proliferation in vitro.^67^ Consistent with these findings, NMN supplementation improved postnatal alveolar epithelial differentiation and prevented postnatal lethality partially in mice with epithelial-specific mitochondrial ETC complex I deficiency.^30^

Recently, a group has suggested that pathogenic stem cells may play a role in chronic lung disease. Single-cell suspensions of lung explant tissue were plated onto irradiated 3T3-J2 murine embryonic fibroblasts for cloning. While control lungs yield predominantly a single clonal population, explant lungs from patients with chronic lung diseases, including COPD,^68^ IPF,^69^ and CF,^70^ exhibited multiple clonal populations. These diseased lung clonal libraries drove neutrophilic inflammation when xenografted into immunodeficient mice. Questions remain about their biological role in the development of chronic lung disease and how they are regulated.

## Lessons learned from other organ systems

Recent advances in stem/progenitor cell biology in other organ systems have provided valuable insights that can be applied to the field of lung biology, offering promising avenues for therapeutic strategies and a better understanding of lung diseases.

The ISR has emerged as an attractive therapeutic target for neurodegenerative diseases. One relevant approach involves the use of the ISR inhibitor (ISRIB), a small molecule that weakly inhibits the ISR by enhancing eIF2B activity.^33^ Integrated stress response activation reduces global protein translation but paradoxically increases the translation of specific mRNAs of transcription factors such as ATF4, ATF5, CHOP, and GADD34, both of which are improved by ISRIB. Integrated stress response inhibitor has shown efficacy in preclinical models of a variety of neurodegenerative diseases as well as normal aging.^71-79^ Its benefits are now being evaluated in a clinical trial in patients with amyotrophic lateral sclerosis (ALS), one of the most common adult-onset motor neuron diseases (NCT04948645). In iPSC models of ALS in which the disease-associated mutation in vesicle-associated membrane protein–associated protein B (VAPB) P56S was introduced by CRISPR, the mutation caused reduced neuronal firing, mitochondrial dysfunction, and activation of the ISR. Integrated stress response inhibitor improved neuronal firing in iPSC-derived neurons carrying the VAPB P56S mutation.^80^ Notably, ISRIB has also shown efficacy in various mouse models of pulmonary fibrosis^31^^,^^32^ and promoted alveolar epithelial cell differentiation, rescuing postnatal mortality in mice with epithelial mitochondrial complex I deficiency.^30^

Spatial metabolomics, matrix-assisted laser desorption ionization mass spectrometry imaging (MALDI-MSI), has recently emerged as a powerful tool, particularly in cancer research, by providing spatial information of metabolites in fresh frozen tissues at a resolution of 10-50 μm.^81^ Combined with isotope tracing, MALDI-MSI can evaluate the activity of metabolic pathways and quantitatively assess the contribution of nutrients in different tissue regions. For example, using MALDI-MSI with ^13^C in vivo metabolic flux analysis, it was shown that free fatty acids are the major contributors of TCA cycle carbon in the renal medulla, whereas glutamine and citrate are more utilized in the renal cortex.^81^ Given the increasing evidence that mitochondrial metabolism is critical for lung stem/progenitor cell function, the application of spatial metabolomics may provide unique insights into the metabolic heterogeneity within lung tissues.

In the field of gastrointestinal biology research, nerves have been reported to control tissue repair in the gut through cytokine signaling and interaction with immune cells^82^^,^^83^ and to regulate cancer growth.^84^^,^^85^ The mammalian respiratory system also involves the nervous system. For example, the vagus nerve innervates the trachea and bronchi^86^^,^^87^ and contributes to immune function and allergen-induced airway hyperreactivity, in part through rare neuroendocrine cells in the lung, suggesting its biological role in lung health and diseases such as asthma.^88-90^ In addition, a recent study reported that sympathetic neurons promote the growth of small cell lung cancer.^91^ As in the gastrointestinal system, whether neurons and neuroendocrine cells play a role in lung stem/progenitor cell behavior and potentially contribute to disease progression remains an open question that warrants further investigation.

## Conclusions

The workshop concluded with strategic discussions on shifting the research paradigm from studying disease development and progression to uncovering the mechanisms that maintain respiratory health and resilience. This includes establishing standardized metrics of lung health and resilience and understanding the genetic and environmental factors, along with the metabolic and cellular signaling pathways that control lung stem/progenitor cell behavior. Such insights will aid in discovering biomarkers linked to causal mechanisms and identifying therapeutic targets to enhance respiratory health and resilience. Key knowledge gaps and corresponding recommendations are listed in Tables 1 and 2. This will be achieved through continued efforts to foster interdisciplinary collaborations; integrate knowledge from diverse fields such as computational biology, environmental science, immunology, and regenerative medicine; and leverage existing resources.

**Table 1:** Key knowledge gaps in respiratory health and resilience research.

Absence of standardized, quantitative metrics to define and measure respiratory health and resilience Incomplete understanding of stem/progenitor cell populations across all structural cell lineages, including epithelial, endothelial, mesenchymal, and immune compartments Limited mechanistic insight into how lung stem/progenitor cells are regulated and contribute to respiratory health and resilience Technical limitations in experimental models to recapitulate complex human lung biology Lack of validated biomarker panels that reflect respiratory health and resilience Few identified therapeutic targets directly linked to pathways governing stem/progenitor cell resilience

**Table 2:** Recommendations for advancing respiratory health and resilience research.

Shifting the Research Paradigm: From a focus on disease progression to understanding the underlying mechanisms of respiratory health and resilience Standardize metrics to define and measure progenitor cell resilience and lung health. Leverage existing longitudinal studies or design new ones that follow healthy individuals or patients with early stage disease, archiving tissue samples over time. Advance computational modeling to integrate multimodal datasets, enabling a comprehensive understanding of lung function across the lifespan. Increase public awareness and education about the importance of lung health resilience and preventive strategies. Elucidating the Biological Mechanisms Behind Lung Stem/Progenitor Cell Behavior: To identify targets that promote respiratory health and resilience Determine the biological roles of the discretely defined stem/progenitor cell populations in lung health and resilience and link them to functional outcomes related to injury, infection, and repair. Investigate the role of cellular metabolism and epigenetic changes in lung stem/progenitor cell resilience. Determine the role of cell-cell interactions in maintaining lung progenitor resilience including epithelial-immune, epithelial-endothelial, and epithelial-mesenchymal interactions. Understand and dissect the novel function of genetic factors associated with chronic obstructive pulmonary disease and pulmonary fibrosis in human cellular, tissue, and organoid models, as well as animal models. Investigate how the interaction of genetic and environmental factors and genetic interactions among polygenic factors for chronic lung diseases affect the resilience of progenitor cells. Address gaps in our understanding of the long-term effects of environmental exposures and nutrition/lifestyle on lung progenitor cells and lung resilience. New Technologies and Model Systems Leverage advanced organoid models and induced pluripotent stem cells technologies for disease modeling and therapeutic development, particularly targeting genetic susceptibility factors identified in human population studies. Develop imaging techniques to visualize progenitor cell dynamics and resilience in vivo. Use artificial intelligence and machine learning to analyze complex datasets to gain deeper insight into progenitor cell behavior. Recognize the limitations of experimental models that don’t fully recapitulate human lung pathobiology and validate findings in primary human tissues or other animal models that better recapitulate human lung disease. Develop experimental models that better recapitulate human lung physiology and pathobiology. Discovery of Novel Biomarkers Reflecting Causal Mechanisms Develop comprehensive biomarker panels that connect lung progenitor cell function to respiratory health and disease risk. Continue efforts to identify biomarkers that can be measured peripherally (eg, blood, nasal swab), without access to lung tissue, that reflect the status of lung progenitor cells and are associated with lung function and disease risk. Therapeutic Strategies Pursue the development of pharmacological agents or biologics that specifically target the pathways that regulate progenitor cells to enhance lung resilience. Leveraging Multidisciplinary Insights Encourage interdisciplinary collaborations to enrich research on the mechanisms of lung disease and resilience. Strengthen partnerships across basic science, clinical research, and industry for a comprehensive approach to lung health and disease prevention. Leverage Existing Resources Encourage research community to use existing cohort studies and datasets (eg, NHLBI-funded Lung Health Cohort, SOURCE study) to understand lung resilience and disease onset. Encourage research community to use available resources (eg, NHLBI-funded Molecular Atlas of Lung Development Program (LungMAP), Center for Regenerative Medicine (CReM) of Boston University) to understand functionality of progenitor cells in lung resilience.

## Supplementary Material

aanag107_Supplementary_Data
